# Supplementary material for: Risk factors and true prevalence of bovine tuberculosis in Bangladesh
Source: PLoS One. 2021 Feb 26;16(2):e0247838. doi: 10.1371/journal.pone.0247838 (PMC7909650; doi:10.1371/journal.pone.0247838)
Supplement: S5 File — (DOCX) [file pone.0247838.s005.docx]

Supplementary file 5. Univariable association of independent variables with herd level bovine tuberculosis in Mymensingh district, Bangladesh

| Variable | Category | Positive/Tested  (n=189) | Prevalence (%)  (95% CI) | Chi square  P-value |
| --- | --- | --- | --- | --- |
| Herd size |  |  |  | 0.002 |
|  | ≤ 4 | 6/120 | 5..0 (2.0–11.0) |  |
|  | > 4 | 14/69 | 20.3 (11.9–32.0) |  |
| Other animal in herd |  |  |  | 0.05 |
|  | No | 4/55 | 7.3 (2.4–18.4) |  |
|  | Buffalo, goat and sheep | 5/80 | 6.2 (2.3–14.6) |  |
|  | Dog and cat | 5/25 | 20.0 (7.6–41.3) |  |
|  | Chicken | 6/29 | 20.7 (8.7–40.3) |  |
| Management system |  |  |  | <0.001 |
|  | Semi-extensive | 11/169 | 6.5 (3.5–11.6) |  |
|  | Semi-intensive | 9/20 | 45.0 (23.8–67.9) |  |
| Floor type |  |  |  | 0.21 |
|  | Brick floor | 9/114 | 7.9 (3.9–14.9) |  |
|  | Mud floor | 11/75 | 14.7 (7.9–25.2) |  |
| Grazing |  |  |  | 0.006 |
|  | No | 13/66 | 19.7 (11.3–31.7) |  |
|  | Yes | 7/123 | 5.7 (2.5–11.8) |  |
| New purchase |  |  |  | 0.28 |
|  | No | 10/120 | 8.3 (4.3–15.2) |  |
|  | Yes | 10/69 | 14.5 (7.5–25.5) |  |
| Use of mosquito net |  |  |  | 0.06 |
|  | No | 18/130 | 13.8 (8.6–21.2) |  |
|  | Yes | 2/59 | 3.4 (0.6–12.7) |  |
