# Supplementary material for: Risk factors and true prevalence of bovine tuberculosis in Bangladesh
Source: PLoS One. 2021 Feb 26;16(2):e0247838. doi: 10.1371/journal.pone.0247838 (PMC7909650; doi:10.1371/journal.pone.0247838)
Supplement: S6 File — (DOCX) [file pone.0247838.s006.docx]

Supplementary file 6. Univariable association of independent variables with animal level bovine tuberculosis in Mymensingh district, Bangladesh

| Variable | Category | Positive/Tested  (n=510) | Prevalence (%)  (95% CI) | Chi-square  P-value |
| --- | --- | --- | --- | --- |
| Age (years) |  |  |  | 0.78 |
|  | ≥ 3.25 | 9/128 | 7.0 (3.5–13.2) |  |
|  | >3.25 to ≤ 5 | 12/143 | 8.4 (4.6–14,5) |  |
|  | >5 to 8 | 11/140 | 7.9 (4.2–13.9) |  |
|  | > 8 | 5/99 | 5.1 (1.9–11.9) |  |
| Breed |  |  |  | 0.05 |
|  | Indigenous | 5/149 | 3.3 (1.2–8.1) |  |
|  | Crssbred | 32/361 | 8.9 (6.2–12.4) |  |
| Body weight (Kg) |  |  |  | 0.71 |
|  | ≤ 250 | 18/270 | 6.7 (4.1–10.5) |  |
|  | > 250 | 19/240 | 7.9 (4.9–12.3) |  |
| Body Condition Score (1–5) |  |  |  | 1 |
|  | ≤ 2 | 25/349 | 7.2 (4.8–10.5) |  |
|  | >2 | 12/161 | 7.5 (4.1–12.9) |  |
| Pregnancy |  |  |  | 0.49 |
|  | No | 26/325 | 8.0 (5.4–11.6) |  |
|  | Yes | 11/185 | 5.9 (3.2–10.7) |  |
| Parity |  |  |  | 1 |
|  | 0–2 | 22/307 | 7.2 (4.6–10.8) |  |
|  | >2 | 15/203 | 7.4 (4.3–12.1) |  |
| Lactating |  |  |  | 0.82 |
|  | No | 10/153 | 6.5 (3.4–12.0) |  |
|  | Yes | 27/357 | 7.6 (5.1–10.9) |  |
| Coughing |  |  |  |  |
|  | No | 17/387 | 4.4 (2.7–7.1) | <0.001 |
|  | Yes | 20/123 | 15.4 (9.7–23.3) |  |
