# Supplementary material for: Risk factors and true prevalence of bovine tuberculosis in Bangladesh
Source: PLoS One. 2021 Feb 26;16(2):e0247838. doi: 10.1371/journal.pone.0247838 (PMC7909650; doi:10.1371/journal.pone.0247838)
Supplement: Attachment — Submitted filename: Response to Reviewers.docx [file pone.0247838.s007.docx]

**Response to reviewers and editor’s comments**

We thank both of the reviewers and editor for their valuable comments which has led to an improved manuscript. The changes we made have been shown in blue font.

Reviewer #1: Nazimul Islam et al in their study entitled "Bovine tuberculosis true prevalence and risk factors in Bangladesh" have discussed several epidemiological parameters and risk factor which are associated with the bovine tuberculosis in their region

I have following observation / concerns which need attention by the author before this paper can be considered for the publication

1. Whether author examined sero prevelance of M. paratuberculosis in their cohort.

Response: We did not examine seroprevalence of M. paratuberculosis in the skin test positive animals. It could provide information about the cross-reaction of M. paratuberculosis among skin test positive animals. As per OIE guideline, initially we screened all selected animals by caudal fold tuberculin test [CFT] (test positive results may include responses due to M. paratuberculosis also) using bovine purified protein derivatives [PPD] then CFT positive animals were further confirmed by comparative cervical tuberculin test [using both avian and bovine PPDs] which excluded responses from M. paratuberculosis. Moreover, we used serial interpretation of the diagnostic tests which decreases false positive results and hence increases the positive predictive value of the diagnostic tests. We have discussed this in the revised manuscript. Lines: 276-284.

2. It would be advantageous if blood hematogram and immune cells analysis is done to corelate with the potential reason of sensitivity.

Response: Blood hematogram and immune cells analysis were not performed. Immune cell analysis is costly and requires sophisticated laboratory facilities which lack in resource limited settings like Bangladesh. Skin tests are routinely used in Bangladesh for the diagnosis of bovine tuberculosis which is an OIE recommended test for international trade also.

3. Adding some description of immune genetic aspect (MHC gene e.g. ) would also help in understanding the reason of sensitivty of the animals for BTB.

Response: We have added some description of the immunogenetic aspectic of bTB susceptibility in the revised manuscript. Lines: 303-308.

Reviewer #2: The authors in the manuscript planned to determine the true prevalence and identify risk factors for bTB at the animal- and herd-level in Bangladesh. Considering the endemic nature and economic consequences of bovine TB, this work has potential to determine the true prevalence so that effective interventional strategies can be put in place. A major issue is that there has been a recent related publication from same university as mentioned below, though not referred in the manuscript. Authors need to be wary of the overlapping results and observations.
Islam SKS, Rumi TB, Kabir SML, van der Zanden AGM, Kapur V, Rahman AKMA, et al. (2020) Bovine tuberculosis prevalence and risk factors in selected districts of Bangladesh. PLoS ONE 15(11): e0241717. <https://doi.org/10.1371/journal.pone.0241717>

Response: During submission of this manuscript the above mentioned manuscript was not published and hence not cited. Now, in the revised manuscript we have checked for any overlapping results and observation and cited [47]. Lines 274-276 and lines 292-293.

There are some minor changes that need to be incorporated as mentioned below
The Affiliation no. 5 has not been assigned to any author.

Response: The affiliation no. 5 has now been assigned to one author.

Please rephrase line 53.

Response: Line 53 rephrased as suggested by the reviewer. Lines: 51-53.

There is large scale discrepancy in CFT and CCT results that may be explained in discussion.

Response: We have now some description of the discrepancy in CFT and CCT results in the revised manuscript. Lines: 266-270.

Result headings need to be more descriptive to include result summary.

Response: I have added some subheadings in the result section.

Characteristics of the test samples may be provided like gender, age and physical conditions etc.

Response: We provided those in the submitted manuscript as two supplementary files [supplementary files 5 and 6]
